# The complete chloroplast genome of *Tibetia liangshanensis* P. C. Li (Leguminosae: Papilionoideae), an endemic species of China

**DOI:** 10.1080/23802359.2021.1935342

**Published:** 2021-06-07

**Authors:** Ying Guo, Hafiz Muhammad Wariss

**Affiliations:** a Yunnan Key Laboratory for Dai and Yi Medicines, Yunnan University of Chinese Medicine, Kunming, PR China; b Lushan Botanical Garden, Chinese Academy of Sciences, Jiujiang, PR China; c Key Laboratory for Plant Diversity and Biogeography of East Asia, Kunming Institute of Botany, Chinese Academy of Sciences, Kunming, PR China

**Keywords:** Plastome, *Tibetia liangshanensis*, IRLC, phylogenetic relationship

## Abstract

The first complete plastid genome (plastome) of a small genus, *Tibetia* (Ali) H. P. Tsui, was sequenced from *T. liangshanensis* P. C. Li. The total genome size of *T. liangshanensis* was 123,372 bp in length, containing 76 protein-coding genes, 30 *tRNAs* genes, and four *rRNAs*. The genome lacked an inverted repeat (IR) region. Its *rpl22* and *rps16* genes were absent and *clpP* gene lost two introns. The overall GC content was 34.68%. Phylogenetic analysis of the plastome of *T. liangshanensis* with other legumes confirmed its phylogenetic position.

*Tibetia* (Ali) H.P. Tsui is an endemic genus of about 5 species distributed in the Himalayan region and the Qinghai-Tibetan Plateau (QTP) of southwestern China. *Tibetia liangshanenis* P.C. Li is an endemic species in the Hengduan Mountains of SW China and previous morphological (Xie et al. 2012) and phylogenetic analysis (Xie et al. 2016) suggest its relationship with several *Tibetia* species and genera in the inverted repeat lacking clade (IRLC) clade in Papilionoideae. In this study, we report the complete chloroplast genome (plastome) of this species, which will provide valuable resources for further studies on genetic diversity, species delimitation within *Tibetia*, and structural plastome and phylogenetic studies in Leguminosae.

Fresh leaves of *T*. *liangshanensis* were collected from Liangshan County, Sichuan province of China (27°50′3.50′′N and 102°33′9.10′′E). The voucher specimens were deposited in the herbarium of Kunming Institute of Botany, Chinese Academy of Sciences (http://www.kun.ac.cn/, Tao Deng, dengtao@mail.kib.ac.cn) under the voucher number Liuj153027. Total genomic DNA (gDNA) was isolated with a modified CTAB protocol (Doyle JJ and Doyle JL 1987) and send to Beijing Genomics Institute (BGI), Shenzhen, China for library construction and sequencing. Illumina HiSeq2000 platform was used for paired-end (PE) reads generation preparation with 2 × 150 bp PE reads. Libraries were size selected for 350 bp inserts. The plastome sequence of the peanut (*Arachis hypogaea* L.) was downloaded from GenBank (NC_026676) and used as the reference plastome. The raw data were filtered and the plastome was assembled following by Zhang et al. (2020). Contigs were then connected into plastome using Bandage Ubuntu dynamic version 8.0 (Wick et al. 2015). Finally, PE reads were mapped to the plastome using Bowtie2 (Langmead and Salzberg 2012) implemented in Geneious version 9.1.4 (Kearse et al. 2012) to determine if there were any differences. Annotation was performed using GeSeq (https://chlorobox.mpimp-golm.mpg.de/cite-geseq.html) (Tillich et al. 2017), coupled with manual adjustment in Geneious. The online tRNAscan-SE Search Service (Schattner et al. 2005) was used to determine tRNAs. The final complete plastomes were deposited into GenBank with accession number MF193597 for *T. liangshanensis.*

The complete plastome of *T*. *liangshanensis* was 123,372 bp in length and showed inverted repeat (IR) lacking plastome structures as other IRLC species (Tillich et al. 2017). It comprised of 110 unique genes including 76 protein-coding genes, 30 *tRNAs*, and four *rRNAs*. The overall GC content was 34.68%. Similar to other IRLC plastomes. *T. liangshanensis* lost *rpl22* and *rps16* gene. In addition, its *clpP* gene lost two introns. Protein coding regions (PCRs) were 66,108 bp long, representing 53.6% of the complete chloroplast genome. Contrary to non-IRLC *Ammopiptanthus mongolicus* (78,945 bp/153,935 bp = 51.0%), the percentage of *T*. *liangshanensis*’s PCR was higher.

A phylogenetic tree was constructed using RAxML version 8.2.12 (Stamatakis 2014) based on 14 plastomes of Fabaceae. *Senna tora* (L.) Roxb. and *Inga leiocalycina* Benth. were used as outgroups. The phylogenetic results showed that *Tibetia liangshanensis* nested within the IRLC and formed a clade with *Astragalus nakaianus* with 100% bootstrap values (Figure 1). The complete plastome of *T. liangshanensis* will provide a valuable basis for further study on genetic diversification, evolution, and plastome pattern of legumes.

**Figure 1.**
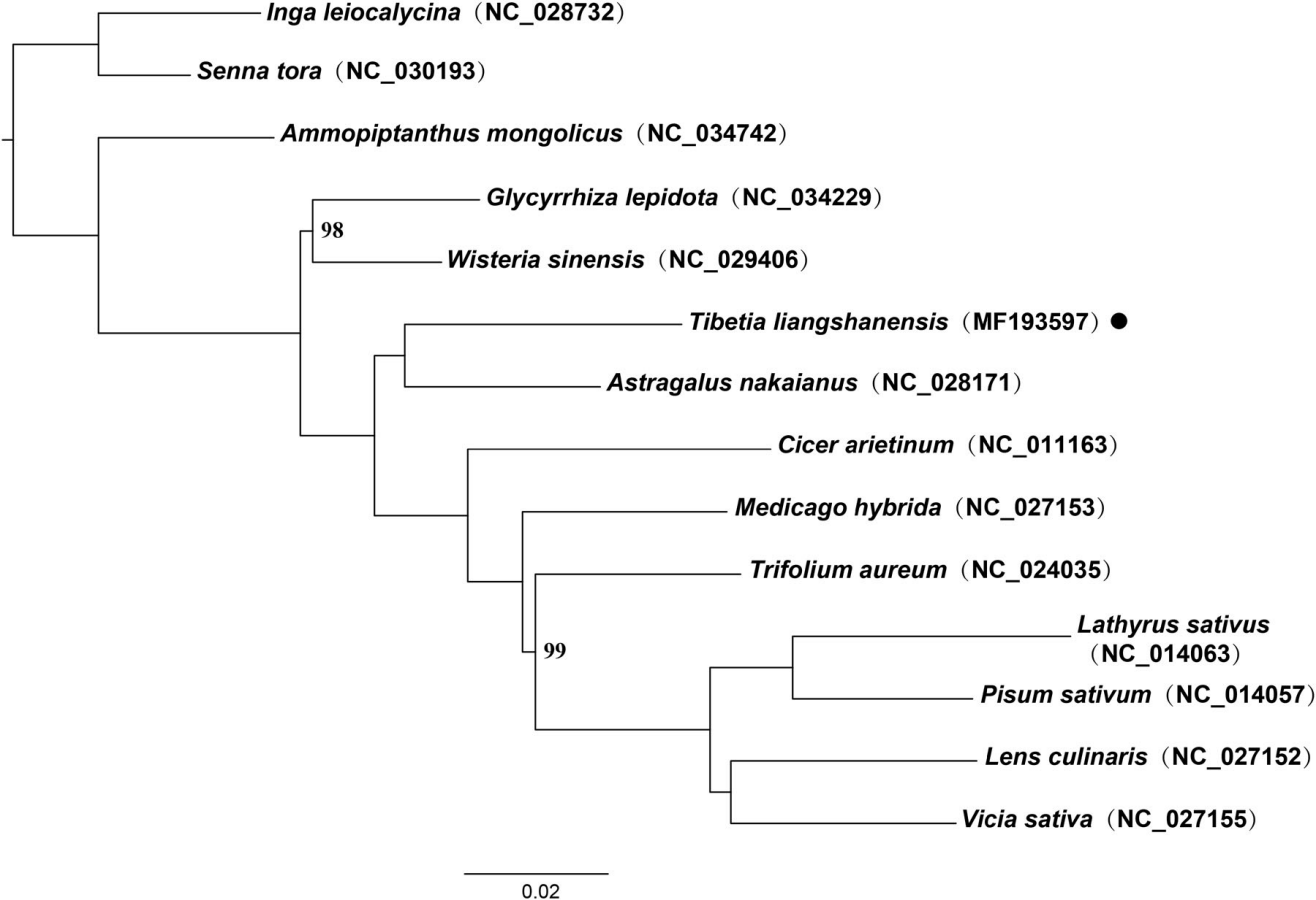
Maximum likelihood (ML) phylogenetic tree based on 14 chloroplast genomes of Fabaceae. ML bootstrap values <100% are shown. The position of *Tibetia liangshanensis* is indicated with black dot.

## Data Availability

The genome sequence data of *T. liangshanensis* are openly available in GenBank of NCBI at [https://www.ncbi.nlm.nih.gov] (https://www.ncbi.nlm.nih.gov/) under the accession no. MF193597, the associated BioProject, SRA, and Bio-Sample numbers are PRJNA725310, SRR14338556, and SAMN18875862, respectively.
